# Antimicrobial resistance, conjugative plasmids and pathogenicity in wastewater and freshwater *Escherichia* spp. in Stockholm, Sweden

**DOI:** 10.1038/s44259-026-00208-5

**Published:** 2026-04-30

**Authors:** Annie Justh de Neczpal, Kaisa Thorell, Laurens Tuts, Geertrui Rasschaert, Alberto J. Martín-Rodríguez, Enrique Joffré, Åsa Sjöling

**Affiliations:** 1 Department of Chemistry and Molecular Biology, CMB, University of Gothenburg, Göteborg, Sweden; 2 Centre for Antibiotic Resistance Research in Gothenburg (CARe), University of Gothenburg, Gothenburg, Sweden; 3 Flanders Research Institute for Agriculture, Fisheries and Food (ILVO), Technology and Food Science Unit, Merelbeke-Melle, Belgium; 4 Department of Microbiology, Tumor and Cell Biology (MTC), Karolinska Institutet, Stockholm, Sweden; 5 Department of Clinical Sciences, University of Las Palmas de Gran Canaria, Las Palmas de Gran Canaria, Spain; 6 Department of Medical Biochemistry and Microbiology, Uppsala University, Uppsala, Sweden

**Keywords:** Microbiology, Environmental sciences

## Abstract

To investigate antibiotic resistance genes (ARGs), conjugative plasmids, and virulence genes in Swedish waterborne *Escherichia* spp., water samples were collected from urban freshwater and Baltic Sea beaches and a primary wastewater treatment plant (WWTP) in Stockholm, Sweden. During the summer of 2022, 68 isolates were recovered using ESBL-selective and non-selective agar, including 40 from wastewater and 28 from fresh or brackish water. Isolates were characterised by phenotypic antibiotic susceptibility testing, conjugation assays, and whole-genome sequencing. Antibiotic residues were quantified, with higher concentrations detected at WWTP inlets and outlets than in natural water sources. Overall, 28 isolates (41.17%) were phenotypically multidrug-resistant (MDR), and 18 (26.47%) carried ≥3 ARGs. WWTP-derived isolates showed a significantly higher prevalence of extended-spectrum β-lactamase (ESBL) genes than freshwater isolates (p < 0.0001). Isolates represented diverse multilocus sequence types (MLST), and most harboured ≥1 plasmid. Sixteen strains transferred conjugative plasmids encoding resistance to cefotaxime, tetracycline, streptomycin, and trimethoprim. ESBL genes, including *bla*_CTX-M-15_, were located on IncF, IncN, IncB/O/K/Z, and IncI plasmids, with IncF plasmids showing lower transfer frequencies than IncN and IncI1 conjugative plasmids. These findings identify WWTPs as a major source of MDR and pathogenic *E. coli*, highlighting their role in environmental dissemination.

## Introduction

Antimicrobial resistance (AMR) in bacteria is recognised as a critical and escalating global health threat^1–3^. The incidence of antibiotic-resistant bacteria (ARB) and multidrug-resistant (MDR) bacteria has increased due to overuse and misuse of antibiotics in healthcare, animal husbandry, industrial waste, and inadequate treatment of diseases^4,5^. Unsafe drinking water sources can cause illness in humans and animals upon consumption, and contaminated or inadequately purified environmental waters and wastewaters may contribute to spread and exposure to microbial pathogens^6–9^. The spread of AMR in pathogenic species has hence emerged as a significant global public health issue due to lack of treatment options.

The increased levels of antibiotic residues found in wastewater, and particularly untreated hospital wastewater, may provide the necessary selective pressure for the retention and spread of antibiotic resistance genes (ARGs)^10,11^. Additionally, the mixture of environmental and pathogenic strains in wastewater treatment plants (WWTP) may allow for the exchange, transfer and/or acquisition of ARGs. The exchange of ARGs between environmental strains and pathogenic strains of clinical importance poses a threat for human health.

*Escherichia coli* (*E. coli*) is typically used as an indicator of faecal pollution in water sources since it is an almost universal commensal resident in the intestines of warm-blooded animals, including humans. Although commensal *E. coli* live in symbiosis with their hosts, pathogenic subspecies of *E. coli* harbouring virulence genes are common causes of intestinal diarrheagenic (DEC) and extraintestinal infections (ExPEC) in humans and animals^12^. DEC are often water- or foodborne pathogens with increased prevalence in natural waters during heavy precipitation and flooding in endemic areas^6,13^. ExPEC are often recovered from sewage, WWTPs or downstream of WWTPs in rivers^14–17^. *E. coli* is also often studied for the presence of ARGs since it is easily isolated and can transfer ARGs through horizontal gene transfer as well as through distribution in anthropogenically influenced water systems, including agricultural areas, urban sewage, and WWTPs.

The growing concern of the emergence of AMR in pathogenic water-borne *E. coli* has resulted in several studies determining the prevalence of MDR bacteria in aquatic environments^15,16,18^. Several studies have established higher levels of ARGs, including extended-spectrum β-lactamases (ESBL), in *E. coli* and other species collected from wastewater samples compared to environmental water isolates^5,16,19^. Although hospital sewage and WWTPs are considered hotspots for the presence of ARB and ARGs^18^, the environment is a significant reservoir and vehicle in zoonotic infections and emerging human pathogens as well as ARB^20^. Antibiotics from urban and agricultural sources persist in soil and water as well as in wastewater and may drive horizontal gene transfer and development of new ARGs and ARB.

The emergence of ESBL-producing strains has been recognised since the 1990s, when mostly TEM/SHV beta-lactamases were described together with emerging *bla*_CTX-M-2_^21^. Since 2000, a dramatic shift in the prevalence of particularly *bla*_CTX-M-15_ has been reported in *E. coli*, where the global expanse is probably mediated by the uropathogenic clone ST131 that predominantly carries *bla*_CTX-M-15_^22^. Wastewater sample studies frequently isolate MDR and ESBL carrying isolates belonging to the ExPEC pathotype. The isolates frequently detected include clonal groups associated with urinary tract infections (UTI) and bloodstream infections (BSI) such as ST131, ST648, ST69, ST95, ST410, ST127, and ST38^17,23–25^.

The present study aimed to investigate the presence of pathogenic and/or MDR *E. coli* isolates and antibiotic residues present in freshwater, brackish water and wastewater sites in Stockholm, Sweden as well as characterise their ability to transfer mobile genetic elements carrying ARGs.

The results show that MDR bacteria with conjugative plasmids carrying ARGs, as well as pathogenic bacteria, were present at multiple sites, which highlights the importance of surveillance and studies of the transmission of ARB and ARGs from the environment back to humans.

## Results

### Genomic analysis of isolates revealed presence of diverse *Escherichia* species across WWTP inlets/outlets and natural water sources in Stockholm, Sweden

**Fig. 1:**
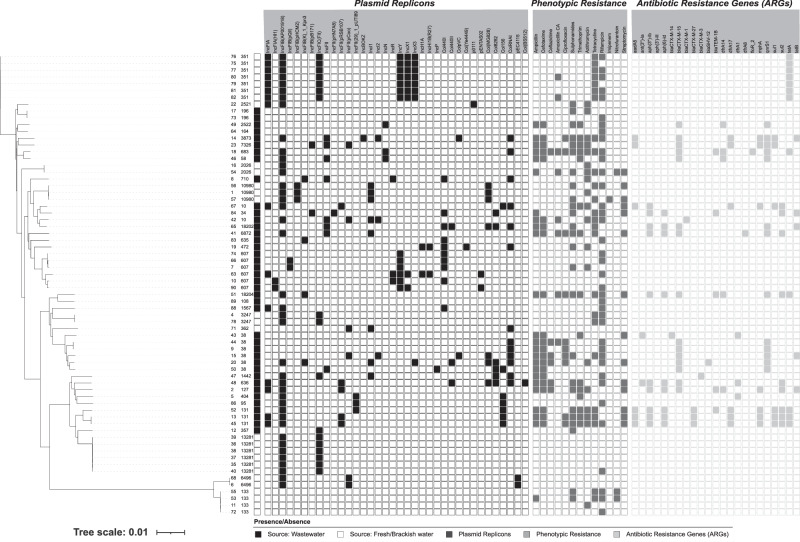
Tree of kmer distance of all *Escherichia* spp. isolates from Stockholm indicating isolation site (WWTP vs fresh/brackish water), plasmid replicons, phenotypic antibiotic-resistance and presence of antibiotic resistance genes (ARGs). The first column represents the isolate number. The second column indicates multi-locus sequence type (MLST). The third column indicates isolation source, where the filled squares indicate isolation from WWTP compared to fresh/brackish water as empty squares. The fourth column indicates presence/absence of plasmid replicons belonging to the plasmid incompatibility groups. The fifth column indicates presence/absence of phenotypic resistance. The sixth and final column indicates presence/absence of antibiotic resistance genes (ARGs). The MLST was determined through the Achtman Scheme; Plasmid Replicon Groups were determined using CGE pipeline PlasmidFinder; Phenotypic Resistance was determined through the disc diffusion method; and the ARGs were determined using CGE pipeline ResFinder. The phylogenetic tree was produced using Mashtree and annotations were added in iTOL.

A total of 68 *Escherichia* spp. isolates were collected from multiple locations (fresh, brackish and wastewater), whereof 40 were wastewater treatment plant (WWTP) isolates (20 isolated from ESBL selective agar, 20 from non-selective agar) and 28 isolates from fresh/brackish water (all isolated from non-selective agar, no colonies were found on ESBL selective agar) (Fig. 1, Table 1 and Supplementary Fig. 1). All isolates were analysed for phenotypic antimicrobial resistance (AMR) by disc diffusion analyses and whole genome sequenced (WGS) followed by analyses for multi-locus sequence type (MLST), antibiotic resistance genes (ARGs), presence of plasmids and virulence genes (Fig. 1, Table 1 and Supplementary Table. 1).

**Table 1:** MLST, total antibiotic resistance gene (ARG) and plasmid content of all 69 *Escherichia* spp. isolates described in this study

Isolate	MLST	Plasmids	Total *n*° ARGs	ARGs								Accession Number
				Aminoglycosides	β-lactams	Tetra-cyclines	Sulfon-amides	Quinolones	Macrolides	Trime-thoprims	Phenolics	
**1. SE-N-W-Ec10**	10980	IncFIB(pHCM2), IncFIB(AP001918), IncI1, Col(MG828)										SAMN48681364
**2. SE-HDO-W-EcE4**	127	IncFIB(AP001918), IncFIA, IncFII(pRSB107)	8	*aph(3”)-lb, aph(6”)-ld*	*bla*_*CTX-M-15*_*, bla*_*TEM-1B*_	*tet(B)*	*sul2*		*mph(A)*	*dfrA14*		SAMN48681365
**4. SE-N-S-Ec1**	3247	IncFIC(FII), IncFIB(AP001918)										SAMN48681366
**5. SE-SI-W-Ec1**	404	IncFIB(AP001918), IncFII(29)_1_pUTI89, Col156	1							*dfrA1*		SAMN48681367
**6. SE-R-W-Ec1**	6496	IncFII(pCoo), IncFIB(AP001918), pEC4115										SAMN48681368
**7. SE-HDI-W-Ec5**	607	IncFIB, IncY, Col440I										SAMN48681369
**8. SE-HDI-W-Ec2**	710	IncFII, IncFIB(K)_1_1_Kpn3, ColRNAI, Col4401, IncR										SAMN48681370
**9. SE-HDO-W-EcE5 (p)**	38	ColRNAI	2		*bla*_*CTX-M-15*_			*qnrS1*				SAMN48681371
**10. SE-HDI-W-Ec8 (p)**	607	IncY, IncFIA(HI1), Col440I, IncR										SAMN48681372
**11. SE-H-S-Ec1-1 (p)**	133											SAMN48681373
**12. SE-SI-W-Ec8 (p)**	357	IncFIC(FII)										SAMN48681374
**13. SE-HD-W-EcE6 (p)**	131	IncFIB(AP001918), IncFII(pRSB107), IncFIA, Col156	9	*aph(3”)-lb, aph(6”)-ld, aadA5*	*bla*_*CTX-M-27*_	*tet(A)*	*sul1, sul2*		*mph(A)*	*dfrA17*		SAMN48681375
**14. SE-SI-W-EcE2 (p)**	3873	IncFII, IncBOKZ, IncI2, IncFIB(AP001918), ColRNAI, ColpVC	7	*aadA5*	*bla*_*CTX-M-15*_	*tet(B)*	*sul1*	*qnrS1*	*mph(A)*	*dfrA18*		SAMN48681376
**15. SE-SI-W-EcE5 (p)**	38	IncI2, IncFII(pCoo), ColRNAI, Col(MG828), ColpVC	3		*bla*_*CTX-M-15*_	*tet(B)*		*qnrS1*				SAMN48681377
**16. SE-R-S-Ec3 (p)**	2026	IncFIB(AP001918)										SAMN48681378
**17. SE-HD-W-Ec7 (p)**	196											SAMN48681379
**18. SE-HDI-W-EcE1 (p)**	683	IncFIB(AP001918), IncN, ColRNAI	6		*bla*_*CTX-M-15*_*, bla*_*TEM-1B*_	*tet(A)*		*qnrS1*		*dfrA14*	*floR_2*	SAMN48681380
**19. SE-HD-W-Ec1 (p)**	472	IncHI1A, IncHI1B(R27), Col(Ye4449), ColRNAI, Col4401										SAMN48681381
**20. SE-SI-W-EcE8 (p)**	38	IncFIB(AP001918), IncFIB(K)_1_Kpn3, IncI1, IncX3, ColRNAI, Col8282, Col(MG828), Col440II	3		*bla*_*CTX-M-15*_*, bla*_*SHV12*_			*qnrS1*				SAMN48681382
**22. SE-N-W-Ec1 (p)**	2521	IncFIA, IncFIB(AP001918), p0111										SAMN48681383
**23. SE-HDO-W-EcE1 (p)**	7326	IncFII(pCoo), IncFIB(pB171), IncFII	6	*aadA5*	*bla*_*CTX-M-15*_		*sul1*	*qnrS1*	*mph(A)*	*dfrA17*		SAMN48681384
**35. SE-N-W-Ec1 COL-R**	13281	IncFIC(FII), IncFIB(AP001918)										SAMN48681385
**36. SE-N-W-E2 COL-R**	13281	IncFIC(FII), IncFIB(AP001918)										SAMN48681386
**37. SE-N-W-Ec3 COL-R**	13281	IncFIC(FII), IncFIB(AP001918)										SAMN48681387
**38. SE-N-W-Ec4 COL-R**	13281	IncFIC(FII), IncFIB(AP001918)										SAMN48681388
**39. SE-N-W-Ec5 COL-R**	13281	IncFIC(FII), IncFIB(AP001918)										SAMN48681389
**40. SE-N-W-Ec6 COL-R**	13281	IncFIC(FII), IncFIB(AP001918)										SAMN48681390
**41. SE-HDI-W-EcE2**	6872	IncI, IncFIB(AP001918), IncFII	6	*aph(3”)-lb, aph(6”)-ld*	*bla*_*CTX-M-15*_		*sul2*	*qnrS1*		*dfrA8*		SAMN48681391
**42. SE-HDI-W-EcE3 (G4)**	10	IncI1, IncI2, IncFIB(AP001918), IncFII, IncFII(pCoo)	1		*bla*_*CTX-M-1*_							SAMN48681392
**43. SE-HDI-W-EcE4**	38		3	*ant(3”)-la*	*bla*_*CTX-M-14*_					*dfrA1*		SAMN48681393
**44. SE-HDI-W-EcE5**	38	ColRNAI	2		*bla*_*CTX-M-15*_			*qnrS1*				SAMN48681394
**45. SE-HDI-W-EcE6**	131	IncFIA, IncFIB(AP001918), IncFII(pRSB107), Col156	9	*aph(3”)-lb, aph(6”)-ld, aadA5*	*bla*_*CTX-M-27*_	*tet(A)*	*sul1, sul2*		*mph(A)*	*dfrA17*		SAMN48681395
**46. SE-HDI-W-EcE7**	58	IncN, IncFII, IncFIB(AP001918)	3		*bla*_*CTX-M-15*_			*qnrS1*		*dfrA14*		SAMN48681396
**47. SE-SI-W-EcE3**	1442	IncFIB(AP001918), IncFIC(FII), IncI1, Col8282, ColRNAI	1		*bla*_*CTX-M-3*_							SAMN48681397
**48. SE-SI-W-EcE4**	636	IncFIA, IncFIB(AP001918), IncFII(pRSB107), Col440II, Col(MG828), Col8282, ColRNAI, Col(BS512)	6	*aph(3”)-lb, aph(6”)-ld, ant(3”)-la*	*bla*_*CTX-M-15*_		*sul2*			*dfrA1*		SAMN48681398
**49. SE-SI-W-EcE1**	2522	IncN	3		*bla*_*CTX-M-15*_			*qnrS1*		*dfrA14*		SAMN48681399
**50. SE-SI-W-EcE6**	38	IncFII, IncP1, Col156, Col8282	2		*bla*_*CTX-M-15*_			*qnrS1*				SAMN48681400
**51. SE-SI-W-EcE7**	18204	IncFIB(K)_1_Kpn3, IncFIB(AP001918)	8	*aph(3”)-lb, aph(6”)-ld*	*bla*_*CTX-M-15*_*, bla*_*TEM-1B*_	*tet(A)*	*sul2*	*qnrS1*		*dfrA14*		SAMN48681401
**52. SE-HDO-W-EcE8**	131	IncFIB(AP001918), IncFII(29)_1_pUTI89, Col156	10	*aph(3”)-lb, aph(6”)-ld, aadA5*	*bla*_*CTX-M-27*_*, bla*_*TEM-1B*_	*tet(A)*	*sul1, sul2*		*mph(A)*	*dfrA17*		SAMN48681402
**53. SE-H-S-Ec1**	133											SAMN48681403
**54. SE-H-S-Tc-CTX2**	2026	IncFIB(AP001918)										SAMN48681404
**55. SE-H-S-Tc-CTX3**	133											SAMN48681405
**56. 1SE-N-W-Ec2**	10980	IncFIB(pHCM2), IncFIB(AP001918), IncI1, Col(MG828)										SAMN48681406
**57. 1SE-N-W-Ec11**	10980	IncFIB(pHCM2), IncFIB(AP001918), IncI1, Col(MG828)										SAMN48681407
**63. SE-HDI-W-Ec1**	607	IncFIA(HI1), IncY, IncX1, IncR, IncHI1A, IncHI1B(R27), pENTAS02										SAMN48681409
**64. SE-HDI-W-Ec3**	164											SAMN48681410
**65. SE-HDI-W-Ec4**	18202	IncFII, Col440I, Col440II, ColRNAI, Col8282, Col(MG828)	5	*aph(3”)-lb, aph(6”)-ld*	*bla*_*CTX-M-15*_	*tet(B)*		*qnrS1*				SAMN48681411
**66. SE-HDI-W-Ec6**	607	IncFIB(pQil), IncY, Col440I										SAMN48681412
**67. SE-HDI-W-Ec7**	10	IncFIA, IncFIB(AP001918), IncFII(pRSB107), Col156, ColRNAI	6	*aph(3”)-lb, aadA5*			*sul1, sul2*		*mph(A)*	*dfrA17*		SAMN48681413
**68. SE-R1-W-Ec2**	6496	IncFIB(AP001918), IncFII(pCoo), pEC4115										SAMN48681414
**71. SE-R1-S-Ec4**	362	IncFII(pCoo), IncI1, ColRNAI										SAMN48681415
**72. SE-H-S-Ec1-1**	133											SAMN48681416
**73. SE-HDO-Ec7**	196											SAMN48681417
**74. SE-HDO-Ec8**	607	IncY, Col440I										SAMN48681418
**75. 1SE-T-W-Ec1**	351	IncFIA, IncFIB(AP001918), IncFIC(FII), IncX1, IncX3, IncY	1			*tet(A)*						SAMN48681419
**76. 1SE-T-W-Ec2**	351	IncFIA, IncFIB(AP001918), IncFIC(FII), IncX1, IncX3, IncY	1			*tet(A)*						SAMN48681420
**77. 1SE-T-W-Ec3**	351	IncFIA, IncFIB(AP001918), IncFIC(FII), IncX1, IncX3, IncY	1			*tet(A)*						SAMN48681421
**78. 1SE-N-S-Ec1**	3247	IncFIB(AP001918), IncFIC(FII)										SAMN48681422
**79. 1SE-T-W-Ec4**	351	IncFIA, IncFIB(AP001918), IncFIC(FII), IncX1, IncX3, IncY	1			*tet(A)*						SAMN48681423
**80. 1SE-T-W-Ec5**	351	IncFIA, IncFIB(AP001918), IncFIC(FII), IncX1, IncX3, IncY	1			*tet(A)*						SAMN48681424
**81. 1SE-T-W-Ec6**	351	IncFIA, IncFIB(AP001918), IncFIC(FII), IncX1, IncX3, IncY	1			*tet(A)*						SAMN48681425
**82. 1SE-T-W-Ec7**	351	IncFIA, IncFIB(AP001918), IncFIC(FII), IncX1, IncX3, IncY	1			*tet(A)*						SAMN48681426
**83. SE-HDO-W-Ec3**	635	IncFIB(K)_1_Kpn3, Col440I										SAMN48681427
**84. SE-HDO-W-Ec4**	34	IncFIB(pB171), IncFII(pHN7A8), Col8282, Col440I	5	*aph(3”)-lb, aph(6”)-ld*	*bla*_*TEM-1B*_	*tet(B)*	*sul2*					SAMN48681428
**86. SE-HDO-W-Ec6**	95	IncFIB(AP001918), IncFII(29)_1_pUTI89, Col156										SAMN48681429
**88. SE-SI-W-Ec3**	1567	IncFIA, IncFIB(AP001918), Col440I										SAMN48681430
**89. SE-SI-W-Ec4**	108											SAMN48681431
**90. SE-SI-W-Ec5**	607	IncY, IncX1, IncFIA(HI1), pENTAS02										SAMN48681432

The first column describes the isolate (the number which the isolate is referred to as throughout the paper, and the code which describes its origin where SE= Sweden; the following letters correspond to isolation source (Supplementary Fig. 1): Nynäshamn (N), Rålambshovsparken (R), Brunnsviken in Hagaparken (H), Sickla Inlet (SI), Henriksdal Inlet (HDI/HD), Henriksdal Outlet (HDO), Tyresö Fiskarholmen (T); followed by sample type: Water (W) or Sediment (S); and lastly, the isolate number on the plate where Ec isolates originate from non-selective plates and EcE isolates originate from ESBL selective plates); the second column shows the Multi-locus sequence type (MLST); the third column shows all plasmids present in the isolate; the fourth column is a summary of the total number of ARGs found in the isolate; and columns 5–12 indicate the specific ARGs found in the isolate which cause resistance to the different indicated antibiotic classes.

All isolates were typed by MLST using the Achtman system^26^ and a phylogenetic tree was generated (Fig. 1). The MLST typing revealed different ST groups present in the different environments, with no overlap of MLSTs between WWTP and fresh/brackish water isolates.

For fresh/brackish water isolates, the 28 isolates belonged to 11 different MLSTs: ST10980, ST3247, ST6496, ST133, ST2026, ST2521, ST13281, ST6496, ST362, ST351 and ST3247 (Fig. 1, Table 1) where the most frequent MLSTs were ST351 (25%, 7/28) and ST13281 (21.4%, 6/28). Six isolates (ST133 and ST6496) branched out and were identified as *Escherichia marmotae*, previously designated as *Escherichia coli* (*E. coli*) clade V (two from Rålambshovsparken and four from Brunnsviken) (Fig. 1 and Supplementary Fig. 1). Three of these isolates were phenotypically MDR, however the corresponding resistance genes were not identified. The 40 WWTP isolates belonged to 24 different MLSTs (Fig. 1 and Table 1), where the most frequent MLSTs were ST607 (15%, 6/40) and ST38 (15%, 6/40). In addition, three WWTP isolates belonged to ST131, whereof two were isolated from the inlet and one from the outlet.

To determine the plasmid content, the WGS data was analysed by PlasmidFinder. The isolates carried plasmid replicons belonging to Incompatibility (Inc) groups: IncFII, IncFIA, IncFIA(HI1), IncFIB(AP001918), IncFIB(pQil), IncFIBp(pHCM2), IncFIB(K)_1_Kpn3, IncFIB(pB171), IncFIC(FII), IncFII, IncFII(pHN7A8), IncFII(pRSB107), IncFII(pCoo), IncFII(29)_1_pUTI89, IncBOKZ, IncI1, IncI2, IncN, IncR, IncY, IncX1, IncX3, IncH1A, IncHI1B(R27), Col440I, Col440II, ColpVC, Col(Ye4449), p0111, pENTAS02, Col(Mg828), Col8282, Col156, ColRNAI, pEC4115 and Col(BS512) (Fig. 1, Table 1).

### WWTP inlet and outlet samples harbour a higher prevalence of ESBL *E. coli* isolates compared to natural water sources in Stockholm, Sweden

AMR profiles determined by disc diffusion showed that 41.17% (28/68) isolates were phenotypically resistant to ≥3 antibiotics classes (10 classes tested). Of the 68 isolates, 26.47% (18/68) carried ≥3 ARGs. All isolates contained the intrinsic chromosomal *mdf(A)* ARG, however, 52.9% (36/68) of the isolates solely carried *mdf(A)*, the majority of these (52.77%, 19/36) came from fresh/brackish water sites collected close to beaches and recreational areas (Fig. 1, Table 1 and Supplementary Fig. 1). Mdf(A) is a membrane transporter which confers tolerance to alkaline conditions and resistance to several antibiotics^27^.

Of the *E. coli* isolated from WWTP samples (inlet and outlet), 52.5% (21/40) were phenotypically resistant to ≥3 antibiotics, and 45% (18/40) carried ≥3 ARGs (Fig. 1 and Table 1). Of the WWTP isolates, 55% (22/40) were extended-spectrum β-lactamases (ESBL)-carriers, where the most predominantly isolated ARG was *bla*_CTX-M-15_ (68.2%, 15/22), which was either chromosomal or carried on IncN, IncI and IncF plasmids (Table 1). In 22.7% (5/22) of the isolates carrying *bla*_CTX-M_ ARGs, there was co-carriage of *bla*_CTX-M_ and either *bla*_SHV_ or *bla*_TEM_. Overall, the most predominantly found ARGs from WWTP isolates were *bla*_CTX-M-15_ (37.5%, 15/40), *qnrS1* (35%, 14/40), *aph(3”)-lb* (25%, 10/40) and *aph(6”)—ld* (25%, 10/40).

Moreover, the three ST131 wastewater isolates (13, 45, 52) harboured IncF and col156 plasmids. ST131 isolates are a global MDR uropathogenic clonal group which frequently carry *bla*_CTX-M_ genes and the Stockholm ST131 isolates carried the highest number of ARGs ( ≥ 9) among the isolates in this study. All ST131 isolates, apart from *mdf(A)*, which is intrinsic in *E. coli*, also carried *aadA5, aph(3”)-lb, aph(6)-ld, bla*_CTX-M-27_*, dfrA17, mph(A), sul1, sul2* and *tet(A)* (Fig. 1 and Table 1). Isolate 52 also carried *bla*_*TEM-1B*_ and was the isolate with most ARGs (10) identified in this study. Isolate 2 (ST127), carried the third highest number of ARGs (8) including *mdf(A), bla*_CTX-M-15_*, sul2, aph(3”)-Ib, aph(6)-Id, bla*_TEM-1B_*, tet(B), mph(A)* and *dfrA14* (Fig. 1 and Table 1).

In contrast, no ESBL-carrying *E. coli* were identified in the fresh/brackish water samples. However, 25% (7/28) of fresh/brackish water isolates were phenotypically MDR, where the predominant ARGs found were *mdf(A)* (100%) and *tet(A)* (25%, 7/28) (Fig. 1, Table 1 and Supplementary Fig. 2). The analysed wastewater isolates were significantly more often ESBL positive (*p* > 0.0001, Fisher’s exact test) compared to fresh/brackish water isolates, however, there was no significant difference in phenotypic MDR between the groups and it is important to note that total number of colonies on ESBL and non- selective plates, respectively, were not recorded in this study.

The *bla*_CTX-M-15_ gene was the most common ESBL and the most predominantly found ARGs in wastewater were *bla*_CTX-M-15_ and *qnrS1* (Supplementary Fig. 2). Furthermore, the co-occurrence of *bla*_CTX-M-15_ and *qnrS1* was the most common (Supplementary Fig. 2). *bla*_CTX-M-15_ has been showed to be associated with *qnr* genes located close together on conjugative plasmids^28^ which supports the findings of this study.

No carbapenem ARGs were found in the sequenced isolates, although, this does not entail that carbapenemases are not present at the sampling sites as further sampling would be required to accurately measure carbapenem ARG levels. However, one isolate (57) was phenotypically resistant to imipenem (IMP) (Fig. 1), although no *bla*_OXA_ or *bla*_CMY_ genes were found in the annotation. The isolate contained OmpC but lacked OmpF, which may be associated with imipenem resistance^29^.

### Levels of antibiotic residues at WWTP inlets and outlets are higher than in natural water sources in Stockholm, Sweden

Filtered water samples were analysed using UHPLC-MS/MS at ILVO (Belgium) to determine the levels of antibiotic residues^30^. Twelve different residues were detected, including β-lactam antibiotics, quinolones, amphenicols, macrolides, sulphonamides, tetracyclines and rifaximin (Table 2). A maximum of ten different residues were identified in one sample. Low concentrations of antibiotics were detected in natural water sources, whereas the inlets and outlets of the Henriksdal WWTP contained higher levels up to 0.25 µg/L (Table 2). The highest concentrations were observed at the inlet of the Sickla WWTP and the outlet of the Henriksdal WWTP. A reduction in both the concentration and the number of antibiotics is noticeable between the Sickla inlet and the Henriksdal outlet, however, in Henriksdal outlet 1, the concentration of ciprofloxacin (CIP) is similar to that of Sickla inlet and exceeds the predicted no effect concentration^31^ (PNECs) for CIP (0.064 µg/L) (Table 2).

**Table 2:** Antibiotic residue concentrations that were at least detected once in water samples collected from freshwater, brackish water and wastewater in Stockholm, Sweden

		Antibiotics residues detected (µg/L)											
Location	Type of water	CIP	TYLV	CHL	CLA	SMX	SPY	TMP	TET	OTC	OFL	CFR	RIFX
**Brunnsviken i Hagaparken**	Freshwater (urban)			<0.01					0.058				
**Rålambshovsparken**	Freshwater (urban)			<0.01					0.058				
**Karlbergskanalen**	Freshwater (urban)	<0.1	0.047										
**Tyresö (Fiskarholmen)**	Brackish water			<0.01									
**Nynäshamn**	Brackish water			<0.01									
**Henriksdal outlet 1**	Wastewater	1.1			0.038	0.064	0.245	0.087					
**Henriksdal outlet 2**	Wastewater	0.015			0.04	0.081	0.179	0.108					
**Sickla inlet**	Wastewater	1.335			0.038	0.345	0.566	0.12	0.298	<0.038	0.044	<0.019	<0.011

Ciprofloxacin (CIP), Tylvalosine (TYLV), Chloramphenicol (CHL), Clarithromycin (CLA), Sulfamethoxazole (SMX), Sulfapyridine (SPY), Trimethoprim (TMP), Tetracycline (TET), Oxytetracycline (OTC), Ofloxacin (OFL), Cefodroxil (CFR), Rifaximine (RIFX).

The results containing ”<” suggests the residue concentration detected is below the level of detection (LOD), the value shown is the LOD^30^. Blank cells represent no detection of antibiotic residues.

### Conjugative plasmids carrying ARGs are transferred at frequencies ranging from 10^−2^ to 10^−7^

To determine if the ARG-carrying plasmids were conjugative, all isolates with phenotypic resistance to one or more of ciprofloxacin (CIP), tetracycline (TET), cefotaxime (CTX), streptomycin (STR) and trimethoprim (TMP) were tested (*n* = 39). The transfer of conjugative plasmids carrying ARGs from freshwater, brackish water and WWTP-derived *E*. *coli* donors to the recipient strain *E. coli* CV601 occurred at a frequency of 41.03%, where 16 of the 39 *E. coli* conjugated with CV601 (Table 3). Conjugation assays using CTX or TET as selective agents were successful, whereas those using CIP, TMP and STR did not yield transconjugants. However, when CTX or TET were used, the ARG for TMP (*dfrA*) was transferred along with the ARGs for CTX (*bla*_CTX-M_*)* in isolates 18, 46 and 49; the ARGs for STR (*aph(3”)-lb, aph(6”)-ld*), *bla*_TEM_ and the ARG for TET (*tet(B)*) in isolate 84.

**Table 3:** Description of plasmids, antibiotic resistance genes (ARGs) and transfer frequency (TF) of the Transconjugants produced in this study

Trans-conjugant	Plasmids in donor	ARGs in donor	TF	Plasmids transferred	ARGs transferred						Accession numbers
					Amino-glycosides	β-lactams	Tetra-cycline	Sulfon-amides	Quino-lones	Trime-thoprim	
**18-CV601**	IncFIB(AP001918), IncN, ColRNAI	*bla*_CTX-M-15_*, bla*_TEM-1B_*, dfrA14, floR, mdf(A) qnrS1, tet)(A)*.	1.33 × 10^−^^2^ ± 0.00587	IncN		*bla*_CTX-M-15_			*qnrS1*	*dfrA14*	SAMN54758342
**41-CV601**	IncFIB(AP001918), IncFII, IncI1	*aph(3”)-lb, aph(6)-ld, bla*_CTX-M-15_*, dfrA8, mdf(A), qnrS1, sul2*	3.31 × 10^−6^ ± 0.00000124	IncI		*bla*_CTX-M-15_			*qnrS1*		SAMN54758343
**42-CV601**	IncFIB(AP001918), IncFII(pCoo), IncFII, IncI1, IncI2	*bla*_CTX-M-1_*, mdf(A)*	4.97 × 10^−5^ ± 0.0000497	IncI1		*bla*_CTX-M-1_					SAMN54758344
**46-CV601**	IncFIB(AP001918), IncFII, IncN	*bla*_CTX-M-15_*, dfrA14, mdf(A), qnrS1*	1.24 × 10^−3^ ± 0.0000713	IncN		*bla*_CTX-M-15_			*qnrS1*	*dfrA14*	SAMN54758345
**47-CV601**	IncFIB(AP001918), IncFIC(FII), IncI1, ColRNAI, Col8282	*bla*_CTX-M-3_*, mdf(A)*	1.12 × 10^−3^ ± 0.000589	IncI1, Col8282, ColRNAI		*bla*_CTX-M-3_					SAMN54758346
**49-CV601**	IncN	*bla*_CTX-M-15_*, dfrA14, mdf(A), qnrS1*	1.59 × 10^−3^ ± 0.000910	IncN		*bla*_CTX-M-15_			*qnrS1*	*dfrA14*	SAMN54758347
**50-CV601**	IncFII, IncP1, Col8282, Col156	*bla*_CTX-M-15_*, mdf(A), qnrS1*	1.77 × 10^−5^ ± 0.0000112	IncFII, IncP1, Col156		*bla*_CTX-M-15_			*qnrS1*		SAMN54758348
**65-CV601**	IncFII, Col440I, Col440II, ColRNAI, Col8282, Col(MG828)	*aph(3”)-lb,aph(6)-ld, bla*_CTX-M-15_*, mdf(A), qnrS1, tet(B)*	1.16 × 10^−5^ ± 0.00000581	IncFII		*bla*_CTX-M-15_			*qnrS1*		SAMN54758349
**75-CV601**	IncFIA, IncFIB(AP001918), IncFIC(FII), IncY, IncX1, IncX3	*mdf(A), tet(A)*	1.44 × 10^−7^ ± 0.0000000563	IncFIA, IncFIB(AP001918), IncFIC(FII)			*tet(A)*				SAMN54758350
**76-CV601**	IncFIA, IncFIB(AP001918), IncFIC(FII), IncY, IncX1, IncX3	*mdf(A), tet(A)*	1.44 × 10^−7^ ± 0.0000000563	IncFIA, IncFIB(AP001918), IncFIC(FII)			*tet(A)*				SAMN54758351
**77-CV601**	IncFIA, IncFIB(AP001918), IncFIC(FII), IncY, IncX1, IncX3	*mdf(A), tet(A)*	1.44 × 10^−7^ ± 0.0000000997	IncFIA, IncFIB(AP001918), IncFIC(FII)			*tet(A)*				SAMN54758352
**79-CV601**	IncFIA, IncFIB(AP001918), IncFIC(FII), IncY, IncX1, IncX3	*mdf(A), tet(A)*	1.76 × 10^−7^ ± 0.000000142	IncFIA, IncFIB(AP001918), IncFIC(FII)			*tet(A)*				SAMN54758353
**80-CV601**	IncFIA, IncFIB(AP001918), IncFIC(FII), IncY, IncX1, IncX3	*mdf(A), tet(A)*	2.47 × 10^−7^ ± 0.000000228	IncFIA, IncFIB(AP001918), IncFIC(FII)			*tet(A)*				SAMN54758354
**81-CV601**	IncFIA, IncFIB(AP001918), IncFIC(FII), IncY, IncX1, IncX3	*mdf(A), tet(A)*	2.12 × 10^−7^ ± 0.000000118	IncFIA, IncFIB(AP001918), IncFIC(FII)			*tet(A)*				SAMN54758355
**82-CV601**	IncFIA, IncFIB(AP001918), IncFIC(FII), IncY, IncX1, IncX3	*mdf(A), tet(A)*	1.76 × 10^−7^ ± 0.0000000815	IncFIA, IncFIB(AP001918), IncFIC(FII)			*tet(A)*				SAMN54758356
**84-CV601**	IncFIB(pB171), IncFII(pHN7A8), Col8282, Col440I	*aph(3”)-lb, aph(6)-ld, bla*_TEM-1B_*, mdf(A), sul2, tet(B)*	1.99 × 10^−5^ ± 0.0000154	IncFII(pHN7A8)	*aph(3”)-lb, aph(6”)-ld*	*bla*_TEM-1B_	*tet(B)*	*sul2*			SAMN54758357

The columns show the transconjugant name (original donor code – CV601); plasmids and antibiotic resistance genes (ARGs) found in the original donors; transfer frequency (TF); plasmids and ARGs transferred to CV601 from WWTP (18, 41, 42, 46, 47, 49, 50, 65, 84) and Fresh/Brackish water (75, 76, 77, 79, 80, 81, 82) isolates. The TF displayed is the mean with standard deviation.

Of the 68 *Escherichia* spp. isolates, plasmid replicons were not identified in nine isolates (five from fresh/brackish water and four from WWTP), possibly suggesting lack of plasmids in these isolates. Interestingly, one of these isolates (isolate 43, ST38), with the presumed lack of plasmids, carried *ant(3”)-la* and *bla*_CTX-M-14_ on the chromosome. Several other isolates carrying only smaller Col plasmids, such as isolates 9 and 44 (both ST38), also contained *bla*_CTX-M-15_ and *qnrS1*, where presence of these ARGs in the largest contig (contig size: 153 kb and 156 kb, respectively) suggest they are chromosomal. Additionally, in both isolates, the *bla*_CTX-M-15_ and *qnrS1* genes were flanked by transposon genes and genes not commonly found on plasmids (e.g. *araE, ygeI*), highlighting the possibility of chromosome insertion. In line with this, ARG transfer to CV601 did not occur when conjugation was tested with these isolates which further supported that the ESBL resistance was chromosomal. ST38 has previously been reported to frequently harbour ESBL genes in the chromosome^32,33^.

**Fig. 2:**
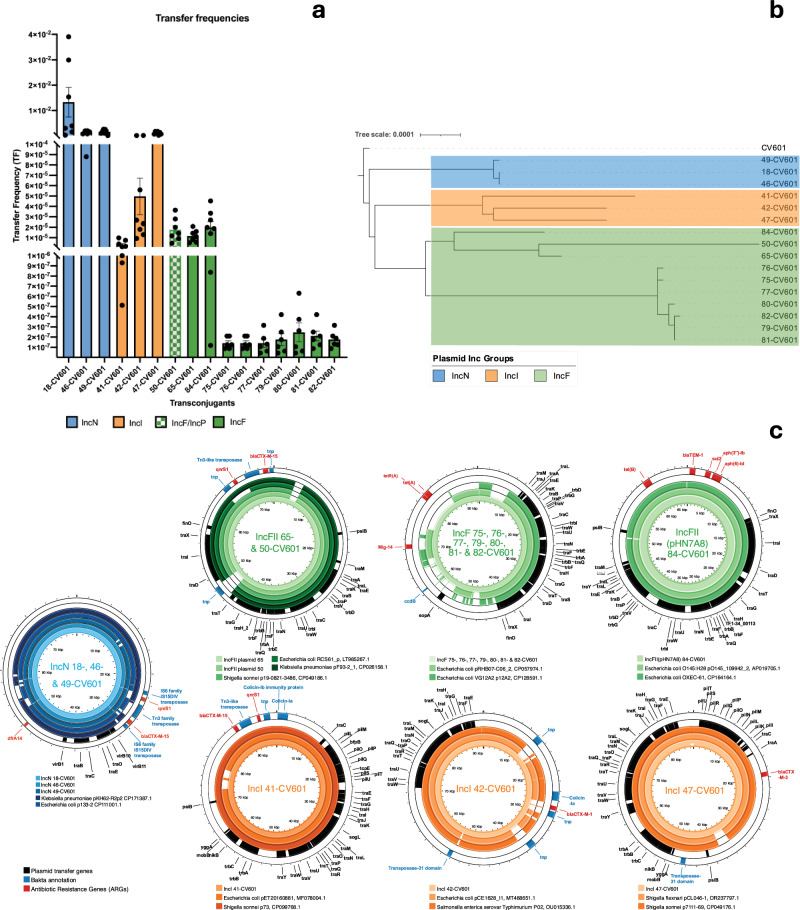
Transfer frequencies (TF), phylogeny and transferred plasmids of all transconjugants. **a** *Transfer frequencies (TFs)* of all obtained transconjugants displayed on a segmented linear axis, including error bars showing the mean. The blue bars indicate the TFs of IncN plasmids, the orange bars indicate IncI TFs, the chequered bar indicates IncF/IncP TF; and the green bars indicate IncF TFs. The graph indicates that the IncN plasmids transferred at the highest TF, where transconjugant 18-CV601 had the highest TF of all transconjugants. The IncN plasmids had higher TFs compared to the IncF plasmids, where the TFs were varied. **b** *Tree of kmer distance of all transconjugants and recipient CV601* showing clustering of transconjugants carrying plasmids of the same Inc group, the IncF plasmids from wastewater isolates cluster differently from the brackish water isolate IncF plasmids, note that transconjugant 50-CV601 branches out due to transfer of two plasmids (IncF and IncP). **c** *Conjugative IncN, IncI and IncF plasmids using alignment of full contigs*. The plasmid figures contain multiple different tracks, the outermost is the antibiotic resistance gene (ARG) track, followed by the Bakta annotation track, genes involved in plasmid transfer track and then the remaining tracks vary between each plasmid, where the backbones are the plasmids discussed in this study, and the remaining tracks are plasmids where sequence comparison using BLAST from sequences derived from GenBank Accession numbers were used. The three IncN plasmids were identical, hence displayed as one plasmid; the three IncI plasmids were not identical, therefore they are displayed as three separate plasmids; one IncP plasmid was isolated but carried no antibiotic resistance genes (ARGs) nor virulence genes and therefore no figure was made; lastly, two IncF plasmids (from 50-CV601 and 65-CV601) were identical, all IncF plasmids isolated from 75-CV601 - 82-CV601 were identical and displayed as one plasmids, and the plasmid from 84-CV601 showed differences compared to all other IncF plasmids. Note that 50-CV601 contained both IncP and IncF plasmids but IncF contained ARGs and represent the reported TF in (**a**). The bar chart was generated using PRISM, the phylogenetic tree was generated using Mashtree and ITOL, the plasmids were generated using Proksee.

Among the donors that transferred ARG-carrying plasmids to CV601, 56.3% (9/16) were wastewater isolates and belonged to ST683, ST6872, ST10, ST58, ST1442, ST2522, ST38, ST34, and ST18202. The transferred wastewater plasmids were IncN (33.3%, 3/9), IncF (22.2%, 2/9) IncF + IncP (11.1% 1/9), and IncI (33.3%, 3/9) (Table 3, and Fig. 2A, B). All carried ESBL ARGs (*bla*_CTX-M_ (88.9%, 8/9) or *bla*_TEM_ (11.1%, 1/9). A subset also co-carried *qnrS1* (60%, 6/9) and *dfrA14* (30%, 3/9), and only one plasmid co-carried *sul, aph* and *tet(B)* ARGs (Table 3). Additionally, isolates 47 and 50 also transferred smaller col plasmids (colRNAI, col8282, col156). The wastewater plasmids had a transfer frequency (TF) range of 10^−2^–10^−7^ (Fig. 2). The IncN plasmids had a higher TF range of 10^−2^–10^−4^ compared to IncF plasmids (10^−5^–10^−7^) (Table 3 and Fig. 2a).

The plasmid with the highest TF (1.33 × 10^−2^) was the IncN plasmid from isolate 18 (18-CV601 transconjugant), which is significantly higher compared to other TFs (*p* < 0.0001, Tukey’s multiple comparisons test). The IncN plasmids transferred from isolates 46 (46-CV601) and 49 (49-CV601) had similar TFs (1.24 × 10^−3^ and 1.59 × 10^−3^, respectively) (Table 3 and Fig. 2). Comparing the content of these three plasmids, there is no genetic variation which could account for the difference in TF. Additionally, upon aligning all three IncN plasmids, they appear almost identical (Fig. 2c). IncI plasmids had the second highest TFs followed by IncF plasmids (Table 3 and Fig. 2a). Furthermore, the three ST131 wastewater isolates (13, 45, 52) did not transfer conjugative plasmids, despite harbouring IncF plasmids.

Forty-one percent (7/16) of the conjugative plasmids originated from fresh/brackish water isolates, in these isolates the only plasmids transferred were IncF plasmids containing *tet(A)* and all donors belonged to ST351 (100%). The transfer frequencies of these plasmids were lower than those originating from wastewater isolates (Table 3 and Fig. 2a). Interestingly, TET ARGs were not transferred by any of the WWTP donors.

IncF plasmids were the only plasmids transferred from both wastewater (IncFII and IncFII(pHN7A8)) and fresh/brackish water (IncFIA, IncFIB(AP001918), IncFIC(FII)) isolates to CV601 (Table 3). The TFs varied where the IncF plasmids from wastewater donors had higher TFs compared to the IncF plasmids from fresh/brackish water donors (Fig. 2a). Additionally, in the isolates which carried both IncN and IncF plasmids (18 and 46), the IncN plasmid seemed to be favoured and transferred through conjugation (Table 3). The same pattern was observed for IncI and IncF, where IncI was prioritised (41 and 47). The donors which transferred IncF plasmids only had IncF plasmids, no IncN or IncI plasmids. The results highlight that most *Escherichia* spp. isolates carry plasmids, not all contain ARGs, and of those that do, a subset conjugate.

IncN, IncI, IncF and IncP plasmids were not the only plasmids transferred to CV601 during conjugation (Table 3). Isolate 47 transferred IncI1, Col8282 and ColRNAI, whereas isolate 50 transferred IncFII, IncP1 and Col156. Isolate 50 also carried a Col8282 plasmid which was not transferred to CV601. Interestingly, three other isolates carried similar Inc plasmids but did not transfer smaller Col plasmids during conjugation where larger IncN or IncF plasmids were transferred: isolates 18 (ColRNAI), 65 (ColRNAI, Col440I, Col440II, Col8282, Col(MG828)) and 84 (Col8282, Col440I).

### Detection of virulent *E. coli* in WWTP inlets/outlets and in natural water sources

Since most of the isolates (60/68) carried plasmids but a subset lacked ARGs, the prevalence of virulence genes associated with human disease was studied (virulence factor database (VFDB) and VirFinder (CGE)). Sequenced transconjugants were analysed and no virulence genes were detected on the transferred plasmids.

The annotations of the sequenced isolate genomes revealed that most of the *E. coli* isolates contained commonly found virulence genes, such as adhesins *fimH* and *fdeC*. However, a subset of isolates (Supplementary Tables 1 and 2) contained genes related to specific *E. coli* pathotype species: EAEC (enteroaggregative *E. coli*), EIEC (enteroinvasive *E. coli*), ExPEC (extra-intestinal *E. coli*), EPEC (enteropathogenic *E. coli*), STEC (shigatoxin producing *E. coli*), ETEC (enterotoxigenic *E. coli*), and UPEC (uropathogenic *E. coli*).

A total of twenty-five different genes coding for adhesins were identified and associated with different pathotypes (Supplementary Table 2). In addition, ten different toxin genes were identified: *sepA, pic, sat*, *sta2 senB, cdt, cnf1*, *hlyA, vat* and *tcpC* (Supplementary Tables 1 and 2). Multiple adhesins were associated with the EAEC pathotype, including *aaiC, aap, aar, aatA, agg4A, aggA, aggR, air/eaeX*, and *eilA*. Wastewater isolates 23 (ST7326) and 84 (ST34) carried multiple of these genes, including the transcription factor *aggR* and toxins *pic* and *sat*, indicating that they are EAEC, however, these isolates did not carry the heat-stable enterotoxin *astA* often associated with the EAEC pathotype (Supplementary Table 1).

Whereas *aufA*, *papA, papB, papC, papG-II, sfaA*, and *yfcV* are associated with the ExPEC pathotype, all these genes plus *afaA*, except *yfcV* are also associated with the UPEC pathotype (Supplementary Tables 1 and 2). This may explain why many isolates expressing these adhesins are frequently isolated from UTI, BSI and uro-sepsis cases, confirming association with both the ExPEC and UPEC pathotypes. The wastewater ST types (isolate 2 (ST127), 5 (ST404), 13 (ST131), 43 (ST38), 45 (ST131), 52 (ST131) and 86 (ST95)) have been associated with UTIs, BSIs and invasive infections^34^, while the fresh/brackish water isolates 36, 37, 38, 39 and 40 (all ST13281) are not yet described in relation to disease to the author’s knowledge. These isolates carried combinations of toxins s*at, senB, vat cnf1, tcpC* and *hlyA* commonly found in ExPEC and UPEC.

Additionally, two genes were found to be associated with the STEC/EPEC pathotypes: *bfpB* (required for formation of type IV bundle-forming pili) and *lpfA* (long polar fimbriae). The *lpfA* gene was found in 48.52% (33/68) of isolates, comparatively, only 5.88% (4/68) of isolates carried *bfpB*. Isolate 14 co-carried both genes, although none of these isolates carried the hallmark virulence gene for shiga-like toxin *stx* (Supplementary Tables 1 and 2).

Three adhesins were associated with the ETEC pathotype: *nfaE* (diffuse adherence fibrillar adhesin gene)*, cfaE* (tip-localised adhesive subunit of colonisation factor antigen I fimbriae CFA/I) and *tia* (cell invasion determinant, also associated with STEC). Isolate 41 carried both *cfaE* and *tia* as well as the *sta2* gene encoding the heat-stable enterotoxin confirming this isolate to be enterotoxigenic *E. coli* (ETEC) (Supplementary Table 1). This isolate is the first ETEC documented in Swedish wastewater, to the authors’ knowledge^35^.

Four genes coding for colicins or microcins were identified: *mchB* (Microcin H47)*, mchC* (MchC protein)*, mchF* (ABC transporter protein MchF) and *cma* (Colicin M). Only isolate 23 carried all *mch* genes, associated with STEC/EPEC pathotypes, whereas three isolates carried *mchF* only. A total of thirteen isolates (18.84%) carried the *cma* gene and there was no co-carriage of the *mch* and *cma* genes (Supplementary Table 2).

The *iss* gene, coding for Increased serum survival protein, was found in 52.94% (36/68) of all isolates, from both wastewater and fresh/brackish water. Carriage of this gene is associated with the ExPEC pathotype. Another gene implicated in serum resistance, *cvaC*, coding for a serum resistance protein was found in only 5.88% (4/68) of all isolates, of which all 5.88% co-carried *iss*. Carriage of *cvaC* is associated with the UPEC pathotype (Supplementary Table 2).

## Discussion

This study characterised conjugative plasmids, identified ARGs as well as investigated the virulence genes carried by *Escherichia* spp. isolated from fresh/brackish water and wastewater sites in Stockholm, Sweden by whole genome sequencing (WGS) of both antibiotic-resistant and non-resistant isolates to compare the *E. coli* diversity found at fresh/brackish water and wastewater sites. Different MLST types and pathotypes were identified in fresh/brackish water and in wastewater, with no overlap of MLST types between the two environments. This might suggest that strain specific adaptation to the different locations exists, which has previously been seen, especially when comparing wastewater and freshwater sites^25,36^. Naturalised wastewater *E. coli*, strains which have evolved to live and survive in WWTPs, are commonly resistant to wastewater treatment and are commonly MDR, carrying multiple ARGs^37,38^.

Antibiotic residues, ARB and ARGs are widespread in wastewater, often considered a hotspot for the dissemination of AMR^39–41^. Release of resistant bacteria via effluent discharge is cause for concern in the surrounding environment, potentially promoting the selection of AMR in natural environments^42–44^.

In line with this, all WWTP water samples in this study contained detectable levels of antibiotics. The physico-chemical properties of the residues and environmental conditions play a role in affecting degradation and adsorption processes^45^ and varying treatment methods in WWTPs impact the breakdown of antibiotics^46^. Henriksdal outlet showed equally high concentrations of antibiotics as Sickla inlet, a trend is therefore observed, where MDR *E. coli* are isolated from WWTPs with detectable levels of antibiotic residues. This implies that the antibiotic selection pressure together with the MDR observed in *E. coli* ST types frequently detected in WWTPs selects for certain pathotypes in WWTPs. The results from this study show that WWTPs can introduce antibiotic residues into the environment. The concentrations of ciprofloxacin (CIP) at Sickla inlet (1.34 µg/L) and Henriksdal outlet (1.1 µg/L) both exceeded the predicted no effect concentration (PNEC) at 0.064 µg/L established by Bengtsson-Palme and Larsson (2016)^31^ implying selection for AMR. Considering this, our finding of high frequencies of co-carriage of *bla*_CTX-M-15_ and *qnrS* suggests that selection pressure by CIP in WWTPs may drive carriage of both *bla*_CTX-M-15_ and *qnrS* in WWTP isolates. Although it has been widely established that β-lactam antibiotics are easily hydrolysed in water^47^, so it is not possible to correlate these residues with ARGs, although they can still be active and drive antibiotic resistance. Regardless, the finding of frequent co-occurrence of *bla*_CTX-M-15_ and *qnrS* is interesting and *bla*_CTX-M-15_ has been shown to be associated with *qnr* genes located close together on conjugative plasmids^28^, which supports the findings of this study.

Previous studies have found low MLST diversity in ESBL-selected and wastewater isolates, and that the most predominant ST type was ST131^25,36,48^. Correlating with the results from this study, previous studies have shown that ExPEC populations, particularly UPEC, are the predominantly isolated *E. coli* pathotype from wastewater and appear to be more capable of surviving wastewater treatment^9,17,49,50^. Zhi et al.^23^ found clinically relevant ST types (ST131, ST95, ST127 and ST640) in wastewater as well as identifying UPEC strains which were found to be almost identical to clinical UPEC strains, whilst also carrying multiple ARGs. The predominantly isolated ESBL-producing MLST in this study was ST38 (*n* = 6) followed by ST131 (*n* = 3). Isolates belonging to ST38 have been reported to be well-known causes of extra-intestinal infections in humans^51^ and are frequently associated with ARGs^32^ and detected in WWTPs as well as in environmental waters^15,52^. Three ST131 isolates were isolated in this study, whereof two from Henriksdal outlet (isolates 13 and 52) and one from the inlet (isolate 45). It has recently been reported that ST131 isolates are increasing in Europe and Sweden and becoming increasingly resistant to last-line antibiotics^53^. This correlates with the findings from this study, where all three ST131 isolates carried nine or more ARGs. In this study, a predominance of MDR and pathogenic ExPEC/UPEC-associated MLST types were found in wastewater. One ETEC was isolated in wastewater inlet samples while no EIEC or shiga-like toxin (stx) genes were found indicating absence of STEC/EHEC, although other virulence genes associated to STEC were identified.

The environment has been shown to play a role in the AMR crisis, where the environment acts as a reservoir of ARGs and ARB where acquired resistance has been driven mostly by anthropogenic use of antibiotics^8,54,55^. In this study, levels of AMR, ARGs, antibiotic residues and virulence genes were expected to be low in the samples and strains isolated from fresh/brackish water sites. The antibiotic residue concentrations at the fresh/brackish water sites were around the limit of detection (LOD) but CIP, chloramphenicol (CHL) and/or tetracycline (TET) were detected at multiple sites. The environment close to WWTP outlets could therefore be affected with an increase in MDR, possibly conjugative and/or pathogenic, *E. coli*. Other studies have shown virulence markers are common in *E. coli* isolated from WWTP effluents^14^. More concerningly, if pathogenic strains possess stress responses which allow for better survival through wastewater treatment, it may be the case that they are better adapted to surviving in the environment surrounding the outlets.

For *E. coli* isolated from fresh/brackish water sources, 75% (21/28) carried several virulence genes associated with ExPEC and UPEC pathotype species, but fewer genes associated with other pathotype species, such as ETEC, EAEC, STEC and fewer virulence genes overall compared to isolates from wastewater. However, since these *E. coli* were isolated from environmental recreational sites, the risk of exposure to humans is higher compared to wastewater. Studies have suggested that water may be an important vehicle of transmission for ExPEC^56^, where UTIs caused by UPEC strains have been linked to exposure to contaminated recreational water^57^. In line with this, a study in the UK found that the prevalence of ExPEC/UPEC ST38, ST3103, ST131 and ST167, and ST1421 carrying *bla*_CTX-M-15_ was increased in river sediment collected downstream of a WWTP^15^. A study of AMR Enterobacteriaceae in rivers and canals in Switzerland identified carbapenemase genes in ExPEC and UPEC- associated STs ST38, ST73, ST167, ST410 and ST648^52^. ST38 is considered a high-risk clone responsible for the spread of *bla*_OXA-48_^52^.

Six *E. marmotae* isolates were identified in freshwater samples located within Stockholm city. *E. marmotae* has been identified as the most prevalent *Escherichia* clade present in the environment and has been found in bird faeces, water and soil^58^. *E. marmotae* has also been reported to infect humans and the isolates carried virulence genes (*astA, hlyF* and *kpsMII_K5)*. These findings indicate that several species of environmental *Escherichia* might be able to cause disease upon exposure to recreational water.

Plasmids have been shown to be the main source of ARGs for *E. coli* isolated from wastewater^59^. Presence of *E. coli* carrying ARG-containing conjugative plasmids in WWTPs is reason for concern as bacterial species are in proximity, risking both inter- and intra-species conjugation, therefore the risk of ARG spread is high.

The present study found high transfer frequencies (TF) of IncN, IncI and IncP plasmids while IncF plasmids had lower TF. Conjugative plasmids’ TF has been shown to differ between plasmid Inc types. IncI plasmids have been reported to spread at rates of 1 × 10^−2^–1 × 10^−4^ during both in vitro and in vivo experiments^60^. IncF plasmids, including plasmids from ST131, have been shown to display low or absent TFs, while IncF transferred from a ST69 donor had a TF of 1 × 10^−4^ ^60^. These results correlate with the results of this study where variations were observed in TF of IncF plasmids, with no transfer of IncF plasmids from ST131 isolates while a TF of 1 × 10^−5^ was recorded in isolates 65 (ST18202) and 84 (ST34). Isolates from sediment and river water downstream of a WWTP found TFs to an *E. coli* recipient between 10^−7^ and 10^−4^ including donors ST131 and ST38^15^. The TF to CV601 for an IncP plasmid has previously been reported to be 1 × 10^−2^ ^61^, while the same author reported TFs of 7.8 × 10^−3^ for an IncFIIY plasmid^62^. Conjugation assays where Bolivian river water microbiota was used as donor found only IncN plasmids^63^. These results are in accordance with studies of transfer from hospital sewage where IncN plasmid transfer to CV601 was found to be common^64^. The results from the conjugation assays in this study suggest that IncN plasmids in general have high TFs which supports multiple findings of IncN plasmids when water microbiota has been used as donor.

High TFs of IncN plasmids has been reported previously^65^. The present study shows an agreement with several studies and indicates that transfer frequencies of IncN, IncP and IncI1 are higher than the commonly found IncF plasmids in *E coli*.

In this study, we also observed that IncN and IncI plasmids were preferentially transferred over IncF plasmids in isolates containing a combination of these plasmids. IncF plasmid transfer inhibition by IncI plasmids has previously been described^66–70^, but there are no reports of IncF transfer inhibition by IncN plasmids. However, transfer inhibition of IncP plasmids by IncN, IncF and IncI plasmids is described in several studies^71–73^. Nevertheless, isolate 50 in this study transferred both IncP and IncF plasmids.

Pallares-Vega et al.^74^ showed that low temperatures and nutrient limitations (environmental conditions) greatly affected TFs of a conjugative IncP plasmid, which highlights that TFs observed in optimal conditions (e.g. 37 °C and high nutrients) may lead to an over estimation of TFs. It was noted that lower nutrients impacted TF more than lower temperatures. However, albeit at a very low level, conjugation still occurred at low temperatures and in nutrient-lacking conditions, which could suggest that conjugation may occur in the environment at low rates. In addition, when considering WWTPs, the temperature is low, but nutrients are often present, which further highlights WWTPs as possible hotspots for conjugation.

*E. coli* isolates carrying ARGs may carry a fitness cost in terms of decreased growth rate, competitive ability and/or virulence^75,76^. In an antibiotic free environment, the cost of carrying ARGs has been shown to be highly variable, ranging from costs of resistance as high as >50%, to little, if any cost^77–80^. Vogwill and MacLean found that AMR by plasmid acquisition confers a smaller cost compared to chromosomal mutations causing AMR. The relatively low cost of carrying an ARG-containing plasmid could explain why plasmids play a principal role in the evolution of resistance^81^. Due to this low cost on fitness, the isolates have no reason to lose the plasmid, highlighting that MDR isolates carrying conjugative ARG plasmids released in effluent from wastewater, are likely to keep the plasmid once they enter the environment, in absence of selective pressures faced in the WWTP.

However, in another more recent study, plasmids carrying multiple ARGs were shown to reduce bacterial fitness by 2.9% per generation^82^. Individually, the ARGs were mostly cost-free, apart from *bla*_CTX-M-15_ and *tetAR*, which were responsible for the entire cost of the plasmid. Therefore, finding isolates in the environment which carry such plasmids might suggest that there are other selective forces acting on environmental MDR *E. coli*.

The risks associated with the spread of clinically relevant ARGs in the environment are not yet fully understood. This study contributes to current knowledge of *E. coli* ecology and AMR by emphasising the importance of investigating both antibiotic-resistant and non-antibiotic-resistant isolates to generate comprehensive and unbiased surveillance data. Furthermore, the findings underscore the importance of integrated surveillance of WWTP effluents and adjacent environmental sites to identify potential increases in pathogenic and/or AMR bacteria. While this study focused on *E. coli* due to its widespread use as an indicator of anthropogenic contamination, it should be noted that ARGs may also be transferred by other bacterial species. Metagenomic studies conducted in the Baltic sea have detected ARGs, including those conferring resistance to beta-lactams, at all sampling locations; although high abundances of Gammaproteobacteria were present in fresh and brackish waters, the prevalence of Enterobacterales was low and *Escherichia* spp. were not detected^83^.

Sampling was conducted at a single time point during the summer season in Sweden, precluding assessment of seasonal variation. As higher prevalences of pathogenic bacteria are globally associated with warm and wet seasons, the present data represent a temporal snapshot and may overestimate pathogen occurrence in the environment surrounding Stockholm, Sweden. Regardless, the results from this study underscore the importance of environmental AMR surveillance, where natural and naturalised *E. coli* may act as ARG reservoirs which, when acquired by virulent and pathogenic isolates, could complicate treatment outcomes.

## Methods

### Isolation of *Escherichia coli* from water

Water samples were collected from five environmental sites in and around Stockholm city in Sweden in 2022. Freshwater samples were collected at Brunnsviken in Hagaparken, Karlbergskanalen, and Rålambshovsparken, all located in Stockholm city, and Brackish water from Tyresö, and Nynäshamn located outside the city (Supplementary Fig. 1). Samples were also collected from four wastewater sites (Henriksdal inlet, outlet, Sickla inlet, and Henriksdal biofilter). At each sampling site, 1.5 l water (W) was collected, and at all sites except the wastewater sites and Karlbergskanalen, 25 ml sediment (w/v) (S) was collected in sterile bottles or tubes and directly transported to the laboratory.

For sediment samples, bacteria were extracted by centrifugation (500 rpm, 4 °C), followed by washing with 25 ml Phosphate Buffered Saline (PBS) and shaking for 1–2 h. Liquid from sediment samples were directly plated on RAPID’E.coli 2 Agar (Biorad) and Thermo Scientific™ Brilliance™ ESBL Agar (37 °C, 24 h) for *Escherichia coli* (*E. coli*) isolation.

For all water samples (fresh/brackish and wastewater), 250 ml was vacuum-filtered using 0.45 µm filters (MF-Millipore^TM^ Membrane Filter HAWP02500). The filtered water was used to dilute the original fresh/brackish and wastewater samples to 10^−1^, 10^−2^. The diluted samples (total volume of 250 ml) were filtered as above, and these filters were placed on the surface of RAPID’*E.coli* 2 and Brilliance^TM^ ESBL Agar (37 °C, 24 h).

Presumptive *E. coli* colonies were re-streaked on RAPID’*E.coli* 2 and Brilliance^TM^ ESBL Agar and confirmed using MALDI-TOF (Bruker). The confirmed isolates were stored at −80 °C in LB broth (Invitrogen^TM^ Luria Broth Base powder 12795084) with 20% glycerol.

### Antibiotic susceptibility testing (AST)

The antibiotic resistance profiles of all isolates were determined using the disc diffusion method. The isolates were grown overnight in Luria Broth (LB) with supplemented antibiotics for the transconjugants at 37 °C, 220 rpm. The cultures were adjusted to OD_600_ = 0.1 before inoculation of 100 µl on Mueller Hinton agar (MH) (Thermo Scientific^TM^ Mueller Hinton Agar dehydrated CM0337B). Antimicrobial susceptibility discs (Oxoid^TM^) were placed on the surface of the MH agar within 10 min of inoculation. The plates were incubated overnight at 37 °C and inhibition halos measured. Breakpoints were measured according to EUCAST clinical breakpoints, following recommended disc concentrations (The European Committee on Antimicrobial Susceptibility Testing. Breakpoint tables for interpretation of MICs and zone diameters. Version 13.0, 2023. http://www.eucast.org).

The antibiotics tested included ampicillin (AMP), cefotaxime (CTX), streptomycin (STREP), ciprofloxacin (CIP), ceftazidime (CAZ), compound sulphonamides (SUL), trimethoprim (TRIM), azithromycin (AZM), norfloxacin (NOR), nalidixic acid (NA), tetracycline (TET), colistin (CT), amoxicillin/clavulanic acid (AMC), rifampicin (RIF), meropenem (MEM), Imipenem (IPM) and nitrofurantoin (F).

### Antibiotic residue analysis

The filtered water samples were frozen upon filtration. Five hundred millilitres of the undiluted filtered water was sent to ILVO, Belgium for antibiotic residue analysis. One hundred millilitres of a water sample was screened for the presence of 78 antibiotic residues from 10 different classes using a UHPLC-MS/MS method validated in compliance with EU Commission Implementing Regulation 2021/808. The analytical procedure involved solid phase extraction using OASIS HLB columns (600 cc, 500 mg), followed by separation in an ACQUITY UPLC H-class (Waters) over a reversed-phase ACQUITY UHPLC BEH C18-column (2.1 × 150 mm;1.7 μm, 100 Å) (Waters), which was coupled to a Xevo TQ-XS spectrometer (Waters) in electrospray ionisation modus. This multiresidue method^30^ enabled the quantification of residues of 20 β-lactams, 22 sulphonamides, 13 (fluoro)quinolones, 9 macrolides, 4 tetracyclines, 4 amphenicols, 2 lincosamides, 2 pleuromutilins, trimethoprim and rifaximin.

### Conjugation assays

Conjugation assays were performed as described previously^63,84^ with minor modifications. *E. coli* donors *n* = 39 (isolated from the fresh/brackish water and wastewater and resistant to selected antibiotics: CTX, CIP, TMP, STP and/or TET) were grown overnight in LB at 37 °C, 220 rpm. The recipient (*E. coli* CV601 (Kan^R^, Rif^R^, Lac^−^, GFP)) was grown overnight in LB with 50 mg/L KAN at 30 °C, 220 rpm^85^.

Aliquots (1 mL) of the donors and recipient were washed in PBS. The aliquots were adjusted to OD_600_ = 1. The donor and recipient strains were mixed in equal volumes (100 µL each) in sterile 1.5 ml centrifuge tubes or 96-well plates to obtain the conjugation mixture. Donors and recipient were also included as controls separately (100 µL of each). All tubes/96-well plates were incubated at 30 °C for 2 h to allow conjugation to occur, which was disrupted by pipetting. The donor, recipient, and conjugation mixture were spotted on LB agar with and without single selection (KAN or antibiotic of choice) and double selection (KAN+ antibiotic of choice) and incubated for up to 48 h at 30 °C. In this study single selection refers to: LB + KAN (200 mg/L) and LB + CTX (5 mg/L) or LB + TET (30 mg/L), and double selection: LB + CTX + KAN or LB + TET + KAN.

Transconjugants were re-streaked on LB agar with double selection as described above, on MacConkey agar (Thermo Scientific^TM^), and disc diffusion was performed as described above to assess transferred resistances. The transconjugants were stored at −80 °C in LB broth with 20% glycerol.

### Calculating conjugation transfer frequencies

The isolates which conjugated in the above assay were selected for further assays to determine transfer frequency. Transfer frequencies were calculated as colony-forming units (CFU) of the transconjugants divided by the CFU of the recipient. Once conjugation ability was confirmed using the method described above, the same method was employed but deviated after disrupting conjugation. The recipient (CV601) was serially diluted to 10^−7^ and the conjugation mixture was serially diluted to 10^−4^. For the recipient (CV601), 100 µL of each dilution was spread on LB + KAN (200 mg/L). For the conjugation mixture, 100 µL of each dilution was spread on the double selective plates (LB + KAN + antibiotic of choice). The donor, recipient, and conjugation mixture were spotted on LB, LB + KAN and LB + KAN+antibiotic of choice as controls. All plates were incubated for up to 24 h at 30 °C. The CFU of the recipient and presumptive transconjugants were counted and the transfer frequency was calculated. This was repeated in biological triplicates with two technical replicates.

### Whole genome sequencing (WGS) and data analysis

Whole genome sequencing (WGS) was performed for all isolates, both donors and transconjugants. Isolates were grown overnight in LB at 37 °C, 220 rpm. Transconjugants were grown overnight in LB supplemented with 200 mg/L KAN and appropriate antibiotic (5 mg/L CTX or 30 mg/L TET) at 37 °C, 220 rpm. Total DNA isolation was performed using DNeasy Blood and Tissue Kits (Qiagen) following the manufacturer's protocol without modification. Isolated DNA was measured using either Qubit™ dsDNA Quantification Assay Kits or Nanodrop (ThermoFisher) and stored at −20 °C. For WGS, all DNA samples were shipped to Eurofins Genomics Europe (Germany) for Illumina paired-end, NovaSeq sequencing (read length: 2 × 150 bp).

Sequencing data was processed using the BACTpipe assembly and annotation pipeline^86^(v. 2.6.1, https://github.com/ctmrbio/BACTpipe)^82^ and re-annotated using Bakta. The presence of ARGs, virulence genes, Multi-locus sequence types (MLST) profiles, and plasmid typing (Inc) was determined using the Centre for Genomic Epidemiology (CGE) pipeline, including ResFinder^87,88^, VirFinder^89,90^, and PlasmidFinder^88,91^. MLSTs were determined using the Achtman scheme^26^. Neighbour-joining trees of k-mer distances were produced using Mashtree^92^ and visualised in ITOL v6^93^. Additionally, MOB-suite^94^, and MOBFinder^95^ were also used to verify the presence of plasmids/plasmid replicons, in addition, contigs were manually curated and plasmid replicons were identified in the same contigs as, for example, ARGs to identify ARG-carrying plasmids. The surrounding genetic environment was also studied to ensure correlation with plasmid or chromosome content. Figures of confirmed plasmids were visualised in Proksee^96^.

### Statistical analyses

The transfer frequencies were analysed in GraphPad Prism using one-way ANOVA and Tukey’s multiple comparisons test. The significance of ESBL presence in wastewater isolates compared to environmental isolates was calculated using Fisher’s exact test.

## Supplementary information

Supplementary Information

## Data Availability

The raw data and assembled genome data presented and discussed in this study can be found on GenBank online repository under bioproject PRJNA1266621. SRA accession numbers: SRR35509390-SRR35509458 and biosample accession numbers: SAMN48681410-SAMN48681432; SAMN48681364–SAMN48681385 and SAMN48681386 – SAMN48681409. The assembled genome data for the transconjugants can be found on GenBank online repository under bioproject PRJNA1347068.
