# Supplementary material for: Antimicrobial resistance, conjugative plasmids and pathogenicity in wastewater and freshwater Escherichia spp. in Stockholm, Sweden
Source: NPJ Antimicrob Resist. 2026 Apr 30;4:32. doi: 10.1038/s44259-026-00208-5 (PMC13133122; doi:10.1038/s44259-026-00208-5)
Supplement: Supplementary file 1 — Supplementary Information [file 44259_2026_208_MOESM1_ESM.pdf]

Supplementary figures

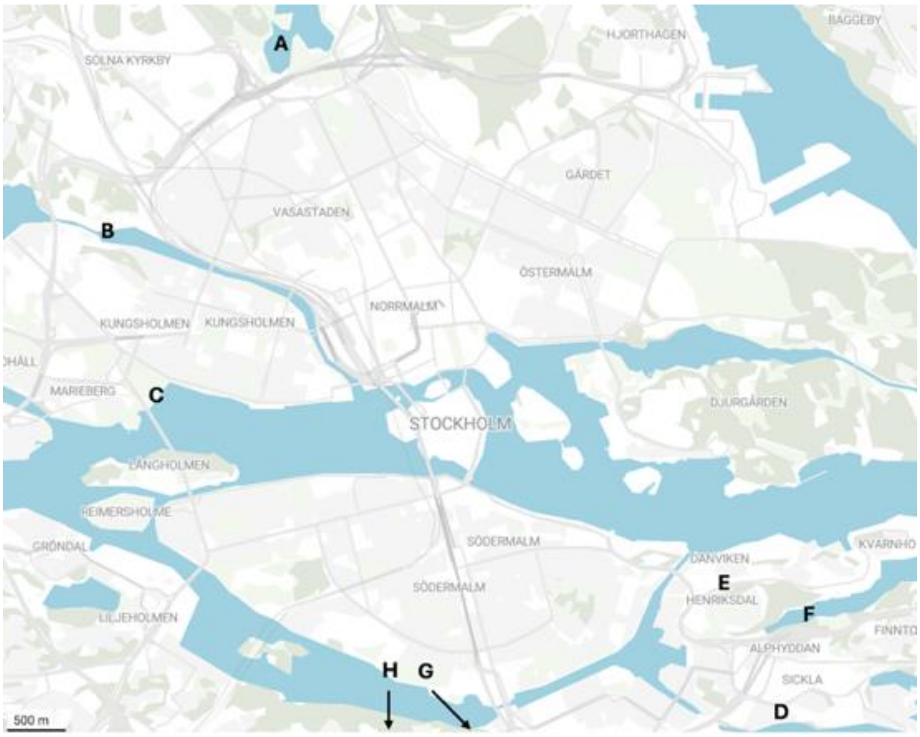

Supplementary Figure 1. Map of water sampling locations in Stockholm, Sweden. Brunnsviken in Hagaparken (A), Karlbergskanalen (B), Rålambshovsparken (C), Sickla Inlet (D), Henriksdal Inlet (E), Henriksdal Outlet (F), Tyresö Fiskarholmen (G), Nynäshamn (H). A, B and C are urban freshwater sites. E, F and D are from the wastewater treatment plant. H and G are not seen on the map, these samples are from brackish water. https://app.datawrapper.de/edit/e5IV1/design

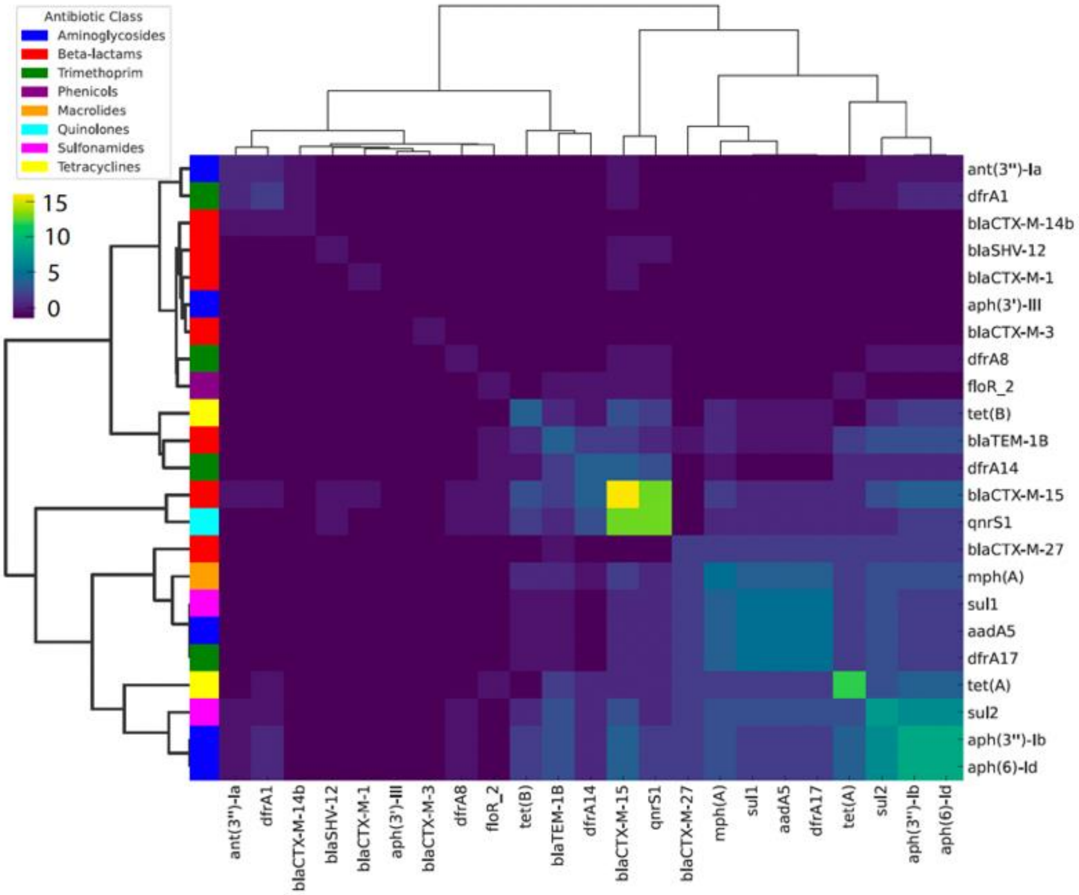

Supplementary Figure 2. Co-occurrence of antibiotic resistance genes (ARGs) in the isolates from Stockholm. Clustering on the y and upper x-axis indicates the isolates involved in the analysis. Colour intensity (blue to yellow) indicates more frequent co-occurrence. The most commonly found ARG was *bla*$_{CTX-M-15}$ and the co-occurrence of *bla*$_{CTX-M-15}$ and *qnrS1* is the most frequent. The *mdf(A)* ARG was removed from this analysis since it is present in all isolates. Correlation was used as the clustering method and the pheatmap R program was used to generate the heatmap.

Supplementary tables

Supplementary Table 1. Virulence genes associated with *Escherichia coli* pathotype species (ETEC, UPEC, STEC, EAEC, EHEC) identified in isolates. The first column describes the isolate (the number which the isolate is referred to as throughout the paper, and the code which describes its origin); the second column shows the Multi-sequence locus type (MLST) and columns 3-9 shows virulence genes present in the isolate belonging to different groups. By examining the annotations, the following genes were found in the isolates: Adhesins (*lpfA, eae, sfaA, iha, nfaE, air, eilA, aap, aatA, agg, aar, afa, PAP, auf, aaiC, tia*), Toxins (*cnf1, vat, senB, pic, astA, tsh, sta2, tcpC, tosA, sat, hlyA, cdt*), Siderophores (*iroN, ireA, aer, iutA, entS, fyuA, ybtP, ybtQ, chuA, iha, sitA*), Serum resistance (*iss, cvaC*), Colicins/Microcins (*mchB, mchC, mchF, cma*), Outer membrane proteins (*ompT*), and additional virulence genes (*capU* and *terC*). EAEC: Enteroaggregative *E. coli*; ETEC: Enterotoxigenic *E. coli;* STEC: Shiga toxin-producing *E. coli*; EPEC: Enteropathogenic *E. coli*; ExPEC: Extraintestinal pathogenic *E. coli*; UPEC: Uropathogenic *E. coli*. Supplementary table 2 describes the virulence genes.

Isolate	MLST	Virulence genes						
		Adhesins	Toxins	Siderophores	Serum resistance	Colicins/ Microcins	Outer membrane proteins	Additional
**1. SE-N-W-Ec10**	10980	*yfcV*		*entS, iroN, sitA*	*iss*	*cma*		
**2. SE-HDO-W-EcE4**	127	*papB, sfaA, aufA, papG-III*	*vat, tcpC*	*fyuA, entS, iroN, ybtP, ybtQ, chuA, sitA*	*iss*	*mchF*	*ompT*	
**4. SE-N-S-Ec1**	3247	*eilA, papB, papC, sfaA, aufA, lpfA,*		*entS, chuA*	*iss*		*ompT*	
**5. SE-Si-W-Ec1**	404	*papA, sfaA, aufA, iha, lpfA*	*sat, senB, vat*	*iutA, fyuA, entS, ybtP, ybtQ, chuA, sitA, iucC, iha*			*ompT*	
**6. SE-R-E-Ec1**	6496	*papB, aufA, lpfA*		*entS, chuA, sitA*			*ompT*	
**7. SE-HDI-W-Ec5**	607	*lpfA, mrk, yfcV*		*entS*				
**8. SE-HDI-E-Ec2**	710	*mrk*		*entS*				
**9. SE-HDO-W-EcE5 (p)**	38	*air/eaeX, eilA, sfaA,*		*entS, chuA*	*iss*		*ompT*	
**10. SE-HDI-W-Ec8 (p)**	607	*lpfA, mrk, yfcV*		*entS*				
**11. SE-H-S-Ec1-1 (p)**	133	*aufA*		*fyuA, entS, ybtP, ybtQ, chuA, sitA*			*ompT*	
**12. SE-SI-W-Ec8 (p)**	357	*sfaA*	*vat*	*fyuA, entS, ybtP, ybtQ, chuA, sitA*	*iss*		*ompT*	
**13. SE-HD-W-EcE6 (p)**	131	*papA, sfaA, iha*	*sat, senB*	*iutA, fyuA, entS, ybtP, ybtQ, chuA, sitA, iucC, iha*	*iss*		*ompT*	
**14. SE-SI-W-EcE2 (p)**	3873	*sfaA, lpfA, bfpB/E, yfcV*		*fyuA, entS, ybtP, ybtQ*				
**15. SE-SI-W-EcE5 (p)**	38	*air/eaeX, eilA, papB, papC, sfaA, bfpB/E*		*entS, chuA, sitA*				
**16. SE-R-S-Ec3 (p)**	2026	*sfaA, lpfA, yfcV*		*entS, iroN, sitA*	*iss, cvaC*	*cma*		
**17. SE-HD-W-Ec7 (p)**	196	*sfaA, lpfA, yfcV*		*entS*				
**18. SE-HDI-W-EcE1 (p)**	683	*sfaA, lpfA, yfcV*		*entS, iroN, sitA*	*iss, cvaC*	*cma*		
**19. SE-HD-W-Ec1 (p)**	472	*sfaA, lpfA, yfcV*		*entS*				
**20. SE-SI-W-EcE8 (p)**	38	*air/EaeX, eilA, sfaA*		*entS, chuA, sitA*				
**22. SE-N-W-Ec1 (p)**	2521	*aggR, sfaA, lpfA, yfcV*		*entS*	*iss*		*ompT*	
**23. SE-HDO-W-EcE1 (p)**	7326	*aggR, aap, aatA, agg4A, sfaA, lpfA, aaiC, yfcV*	*sepA, pic, sat*	*iutA, fyuA, entS, ybtP, ybtQ, iucC*		*mchB, mchC, mchF*		*capU*
**35. SE-N-W-Ec1 COL-R**	13281	*papC, sfaA*	*cdt, vat*	*fyuA, entS, iroN, ,ybtP, ybtQ, chuA, sitA*	*iss*	*cma*	*ompT*	
**36. SE-N-W-E2 COL-R**	13281	*papC, sfaA*	*cdt, vat*	*fyuA, entS, iroN, ,ybtP, ybtQ, chuA, sitA*	*iss*	*cma*	*ompT*	
**37. SE-N-W-Ec3 COL-R**	13281	*papC, sfaA*	*cdt, vat*	*fyuA, entS, iroN, ,ybtP, ybtQ, chuA, sitA*	*iss*	*cma*	*ompT*	
**38. SE-N-W-Ec4 COL-R**	13281	*papC, sfaA*	*cdt, vat*	*fyuA, entS, iroN, ,ybtP, ybtQ, chuA, sitA*	*iss*	*cma*	*ompT*	
**39. SE-N-W-Ec5 (IN/M?) COL-R**	13281	*sfaA*	*cdt, vat*	*fyuA, entS, iroN, ,ybtP, ybtQ, chuA, sitA*	*iss*	*cma*	*ompT*	
**40. SE-N-W-Ec6 (IN/M?) COL-R**	13281	*papC, sfaA*	*cdt, vat*	*fyuA, entS, iroN, ,ybtP, ybtQ, chuA, sitA*	*iss*	*cma*	*ompT*	
**41. SE-HDI-W-EcE2**	6872	*aatA, sfaA, cfaE, bfpB/E, yfcV, tia*	*sta2*	*fyuA, entS, ybtP, ybtQ*				
**42. SE-HDI-W-EcE3 (G4)**	10	*sfaA, bfpB/E, yfcV*		*entS*	*iss*			

**43. SE-HDI-W-EcE4**	38	*air/eaeX, eilA, papG-II, papA, papB, papC, sfaA, iha*	*sat, hylA*	*iutA, fyuA, entS, ybtP, ybtQ, chuA, sitA, iucC, iha*	*iss*			*capU*
**44. SE-HDI-W-EcE5**	38	*air/eaeX, eilA, sfaA*		*entS, chuA*	*iss*		*ompT*	
**45. SE-HDI-W-EcE6**	131	*papA, sfaA, iha*	*sat, senB*	*iutA, fyuA, entS, ybtP, ybtQ, chuA, sitA, iucC, iha*	*iss*		*ompT*	
**46. SE-HDI-W-EcE7**	58	*sfaA, lpfA*		*iutA, entS, iroN, sitA, iucC*	*iss, cvaC*	*mchF*		*capU*
**47. SE-SI-W-EcE3**	1442	*papC, sfaA, aufA, lpfA*	*cdt, vat*	*fyuA, entS, iroN, ybtP, ybtQ, chuA, sitA*	*iss*	*cma*	*ompT*	
**48. SE-SI-W-EcE4**	636	*sfaA, aufA, nfaE, lpfA, afaA*	*vat, tcpC*	*iutA, fyuA, entS, ybtP, ybtQ, chuA, sitA, iucC*			*ompT*	
**49. SE-SI-W-EcE1**	2522	*sfaA, lpfA, yfcV*		*entS*				*capU*
**50. SE-SI-W-EcE6**	38	*air/eaeX, eilA, sfaA*		*entS, chuA, sitA*				
**51. SE-SI-W-EcE7**	18204	*air/eaeX, eilA, sfaA*		*entS, chuA*	*iss*		*ompT*	*capU*
**52. SE-HDO-W-EcE8**	131	*papG-II, papA, papB, papC, sfaA, iha, tia*	*sat, senB, cnf1, hlyA*	*iutA, fyuA, entS, ybtP, ybtQ, chuA, sitA, iucC, iha*			*ompT*	
**53. SE-H-S-Ec1**	133	*aufA*		*fyuA, entS, ybtP, ybtQ, chuA, sitA*			*ompT*	
**54. SE-H-S-Tc-CTX2**	2026	*sfaA, lpfA, yfcV*		*entS, iroN, sitA*	*iss, cvaC*	*cma*		
**55. SE-H-S-Tc-CTX3**	133	*aufA*		*fyuA, entS, ybtP, ybtQ, chuA, sitA*			*ompT*	
**56. 1SE-N-W-Ec2**	10980	*yfcV*		*entS, iroN, sitA*	*iss*	*cma*		
**57. 1SE-N-W-Ec11**	10980	*yfcV*		*entS, iroN, sitA*	*iss*	*cma*		
**63. SE-HDI-W-Ec1**	607	*lpfA, mrk, yfcV*		*entS*				
**64. SE-HDI-W-Ec3**	164	*sfaA, lpfA, yfcV*		*entS*				
**65. SE-HDI-W-Ec4**	18202	*sfaA, yfcV*		*entS, sitA*	*iss*			
**66. SE-HDI-W-Ec6**	607	*lpfA, mrk, yfcV*		*entS*				
**67. SE-HDI-W-Ec7**	10	*yfcV*	*senB*	*fyuA, entS, ybtP, ybtQ*				
**68. SE-R1-W-Ec2**	6496	*papB, aufA, lpfA*		*entS, chuA, sitA*			*ompT*	
**71. SE-R1-S-Ec4**	362	*eilA, papB, sfaA, aufA*	*cdt*	*entS, chuA*			*ompT*	
**72. SE-H-S-Ec1-1**	133	*aufA*		*fyuA, entS, ybtP, ybtQ, chuA, sitA*			*ompT*	
**73. SE-HDO-Ec7**	196	*sfaA, lpfA, yfcV*		*entS*				
**74. SE-HDO-Ec8**	607	*lpfA, mrk, yfcV*		*entS*				
**75. 1SE-T-W-Ec1**	351	*sfaA, lpfA, yfcV*		*entS, sitA*	*iss*		*ompT*	
**76. 1SE-T-W-Ec2**	351	*sfaA, lpfA, yfcV*		*entS, sitA*	*iss*		*ompT*	
**77. 1SE-T-W-Ec3**	351	*sfaA, lpfA, yfcV*		*entS, sitA*	*iss*		*ompT*	
**78. 1SE-N-S-Ec1**	3247	*eilA, papB, papC, sfaA, aufA, lpfA*		*entS, chuA*	*iss*		*ompT*	
**79. 1SE-T-W-Ec4**	351	*sfaA, lpfA, yfcV*		*entS, sitA*	*iss*		*ompT*	
**80. 1SE-T-W-Ec5**	351	*sfaA, lpfA, yfcV*		*entS, sitA*	*iss*		*ompT*	
**81. 1SE-T-W-Ec6**	351	*sfaA, lpfA, yfcV*		*entS, sitA*	*iss*		*ompT*	
**82. 1SE-T-W-Ec7**	351	*sfaA, lpfA, yfcV*		*entS, sitA*	*iss*		*ompT*	
**83. SE-HDO-W-Ec3**	635	*sfaA, lpfA, yfcV*		*entS, sitA*				
**84. SE-HDO-W-Ec4**	34	*aggR, aar, aap, aatA, aggA, aaiC, yfcV*	*pic, sat*	*fyuA, entS, ybtP, ybtQ*		*mchF*		*capU, terC*
**86. SE-HDO-W-Ec6**	95	*papG-II, papA, papB, papC, sfaA, aufA, tia*	*senB, vat, tcpC*	*ireA, fyuA, entS, ybtP, ybtQ, chuA, sitA*	*iss*		*ompT*	
**88. SE-SI-W-Ec3**	1567	*eilA, sfaA*		*entS, chuA*				

**89. SE-SI-W-Ec4**	108	*eilA, sfaA*		*entS, chuA, sitA*	*iss*			
**90. SE-SI-W-Ec5**	607	*lpfA, mrk, yfcV*		*entS*				

Supplementary Table 2. Virulence gene descriptions and prevalence in isolates. The information in the table was collected from different sources[97-99]. The gene name, gene description, which pathotype species the gene is associated with and its occurrence in the isolates is displayed in the first four columns. In the last column, the prevalence in the whole isolate collection is shown. EAEC: Enteroaggregative *E. coli*; ETEC: Enterotoxigenic *E. coli;* STEC: Shiga toxin-producing *E. coli*; EPEC: Enteropathogenic *E. coli*; ExPEC: Extraintestinal pathogenic *E. coli*; UPEC: Uropathogenic *E. coli*.

Gene	Gene Description	Pathotype species	Total number	%/69
aggR	Transcriptional activator	EAEC	4	5.80
aar	AggR-activated regulator	EAEC	1	1.45
aaiC	AaiC, secreted protein	EAEC	2	2.90
aap	Dispersin, antiaggregation protein	EAEC	2	2.90
aatA	Dispersin, transporter protein	EAEC	3	4.35
aggA	AAF/I fimbrial subunit	EAEC	1	1.45
agg4A	AAF/IV fimbrial subunit	EAEC	1	1.45
capU	Hexosyltransferase homolog	EAEC	6	8.70
air/eaeX	Enteroagregative immunoglobulin repeat protein	EAEC	7	10.14
eilA	*Salmonella* HilA homolog	EAEC	12	17.39
sepA	*Shigella* extracellular protease	EAEC/EIEC	1	1.45
pic	Serine protease precursor	EAEC/EIEC	2	2.90
sat	Secreted autotransporter toxin	EAEC/ExPEC	7	10.14
ireA	Siderophore receptor	ExPEC	1	1.45
iss	Increased serum survival	ExPEC	37	53.62
papGII	P-fimbriae	ExPEC	3	4.35
papA	P-fimbriae	ExPEC	6	8.70
papB	P-fimbriae	ExPEC	10	14.49
papC	Pilus-associated protein C	ExPEC	12	17.39
ompT	Outer membrane protease gene	ExPEC	36	52.17
sfaA	S-fimbriae	ExPEC	51	73.91
chuA	*E. coli* heam-utilization gene	ExPEC	33	47.83
iutA	Aerobactin receptor	ExPEC	8	11.59
fyuA	Yersiniabactin receptor	ExPEC	25	36.23
iucC	Aerobactin iron transport system	ExPEC	8	11.59
sitA	Iron caption system, Sit operon	ExPEC	41	59.42
terC	Tellerium ion resistance protein	ExPEC	1	1.45
aufA	Auf-fimbriae (chaperone-usher class fimbrial genes).Associated with Adhesion and Biofilm formation in all types of UTIs.	ExPEC	14	20.29
yfcV	Putative fimbriae	ExPEC	34	49.28
nfaE	Diffuse adherence fibrillar adhesin gene	ETEC	1	1.45
sta2	Heat-stabile enterotoxin ST-I group b	ETEC	1	1.45
cfaE	CFA/I pilus tip adhesin	ETEC	1	1.45
tia	Cell invasion determinant	ETEC/STEC	4	5.80
iha	Adherence protein	STEC	5	7.25

senB	Plasmid encoded enterotoxin	STEC	6	8.70
lpfA	Long polar fimbriae	STEC/EPEC	34	49.28
mchB	Microcin H47 part of colicin H	STEC/EPEC	1	1.45
mchC	MchC protein	STEC/EPEC	1	1.45
mchF	ABC transporter protein MchF	STEC/EPEC	4	5.80
bfpB/E	Bundle-forming pili, outer membrane lipoprotein	STEC/EPEC	4	5.80
afaA	Afimbrial adhesin (adhesion, colonization, has high tropism to kideny) associated with chronic cystitis/pyelonephritis, recurrent cystits/pylenophritis.	UPEC	1	1.45
pap	P-fimbriae (adhesion, colonization, cytokine production, invasion, inflammation, pain, renal tropism, pathogenesis) associated with upper UTIs, acute UTIs, acute pylenophritis, renal failures, acute cystitis.	UPEC	7	10.14
mrk	Type 3 fimbriae (biofilm formation) associated with catheter associated UTIs.	UPEC	7	10.14
sfa	S-fimbriae (adhesion, colonization, dissemination, bacterial ascending factor) associated with meningitis, septicemia and mostly severe upper UTIs.	UPEC	24	34.78
auf	Auf fimbriae chaperone-usher class fimbrial genes (adhesion and biofilm formation) associated with all types of UTIs.	UPEC	14	20.29
iss	Increased serum survival, serum resistance protein (neutralization of anti-bactericidal effect of serum) associated with mostly cystitis and pyelonephritis, bacteremia.	UPEC	37	53.62
cvaC	Serum resistance protein (neutralization of anti-bactericidal effect of serum) associated with mostly cystitis and pyelonephritis, bacteremia.	UPEC	4	5.80
iutA	Siderophore: Aerobactin receptor (growth, iron and hemin uptake system, biofilm formation) associated with severe UTIs.	UPEC	8	11.59
entS	Siderophore (growth, iron uptake) associated with severe UTIs.	UPEC	68	98.55
iroN	Siderophore: Aerobactin receptor or Salmochelin? (growth, iron uptake) associated with severe UTIs.	UPEC	15	21.74
fyuA	Siderophore: Yersiniabactin receptor (growth, iron uptake) associated with severe UTIs.	UPEC	25	36.23
ybtP	Siderophore (growth, iron uptake) associated with severe UTIs.	UPEC	25	36.23
ybtQ	Siderophore (growth, iron uptake) associated with severe UTIs.	UPEC	25	36.23
chuA	Siderophore, Hemin uptake system (biofilm formation, growth and iron uptake) associated with all types of UTIs.	UPEC	33	47.83
ireA	Siderophore, Hemin uptake system (biofilm formation, growth and iron uptake) associated with all types of UTIs.	UPEC	1	1.45
iha	Siderophore, Hemin uptake system (biofilm formation, growth and iron uptake) associated with all types of UTIs.	UPEC	5	7.25
sat	Serine protease autortransporter **toxin** (SAT) (cytotoxic effect on bladder and kidney) associated with mostly pyelonephritis.	UPEC	7	10.14
cdt	Cytolethal Distending **Toxin** (cytolethal factor, human cell apoptotic factor) associated with UTIs.	UPEC	8	11.59
cnf1	Cytotoxic Necrotizing Factor 1 (CNF1) **toxin** (invasion, apoptosis in cell bladder, host cell malfunction) associated with severe UTIs.	UPEC	1	1.45
hlyA	Alpha-hemolysin (**toxin**) (host cell lysis, hemolysis, growth adhesion and inflammation) associated with mostly severe and symptomatic UTIs.	UPEC	2	2.90
vat	Vacuolating autotransporter **toxin** (vat or vactox) (cytotoxic effect on bladder and kidney endothelial tissue) associated with mostly pyelonephritis.	UPEC	12	17.39
tcpC	Toll/interleukin receptor domain containing protein (**toxin**) (bacterial survival, human avoidance system, cytopathic effect on kidney) associated with mostly pyelonephritis	UPEC	3	4.35
papG-III		?	1	1.45
**cma**	Colicin M	?	13	18.84
